# Supplementary material for: Liquid-crystalline behavior on dumbbell-shaped colloids and the observation of chiral blue phases
Source: Nat Commun. 2022 Sep 22;13:5549. doi: 10.1038/s41467-022-33125-y (PMC9500018; doi:10.1038/s41467-022-33125-y)
Supplement: Supplementary file 1 — Supplementary Information [file 41467_2022_33125_MOESM1_ESM.pdf]

Liquid-crystalline behaviour on dumbbell-shaped colloids and the observation of chiral blue phases

Chen *et al.*

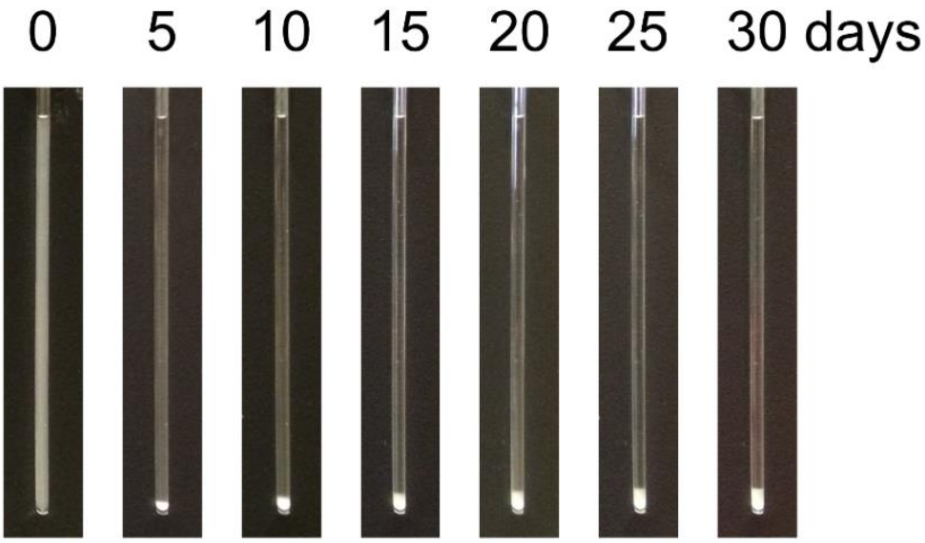

Supplementary Figure 1. Sedimentation of DBCs. Slow sedimentation of DBCs in a capillary tube at different time points.

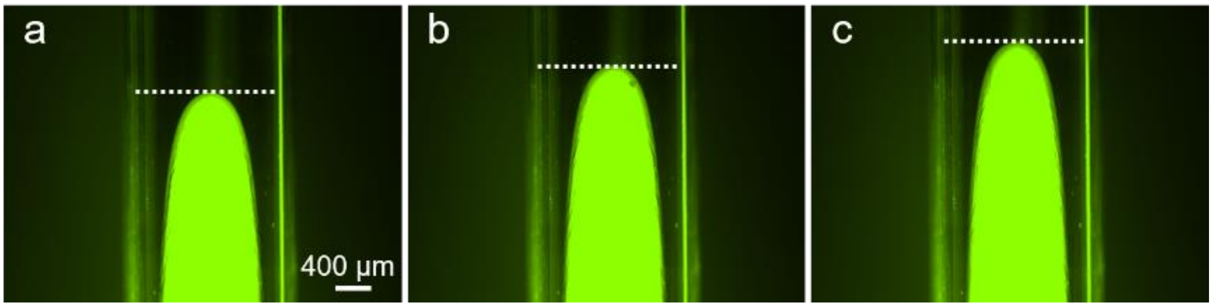

Supplementary Figure 2. A demonstration of the fluidic property of the DBC LC phase. After slow sedimentation of FITC-labelled DBCs in the capillary tube for 30 days, the tube was tilted by 90° to observe the flow of the LC phase with time (time interval: 10 s) under fluorescence microscopy. The white dash lines indicate the flow front of the sediment DBC phase.

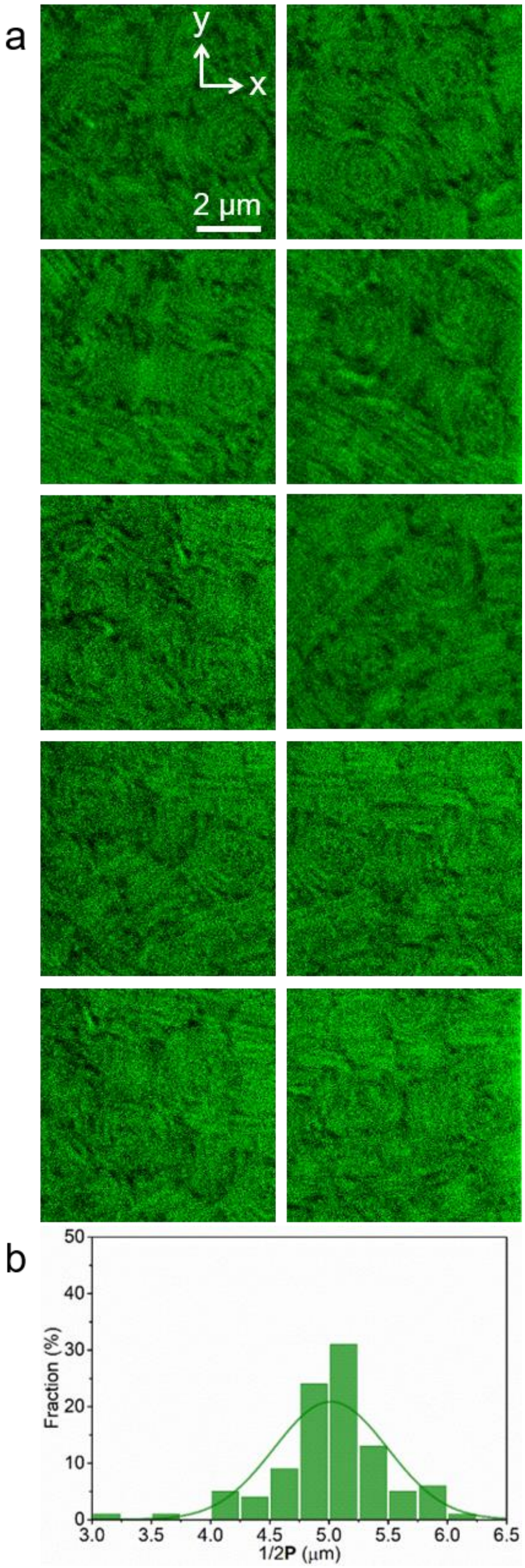

Supplementary Figure 3. Quantification of the $1/2\mathrm{P}$ value by analyzing confocal microscopy images. (a) Ten representative confocal microscopy images of two correlative barrel twist with same handedness assembled from DBCs with L_e = 160 nm, L_c = 1660 nm, D_e = 315 nm, and D_c = 240 nm. (b) $1/2\mathbf{P}$ distribution is obtained by counting 100 columns.

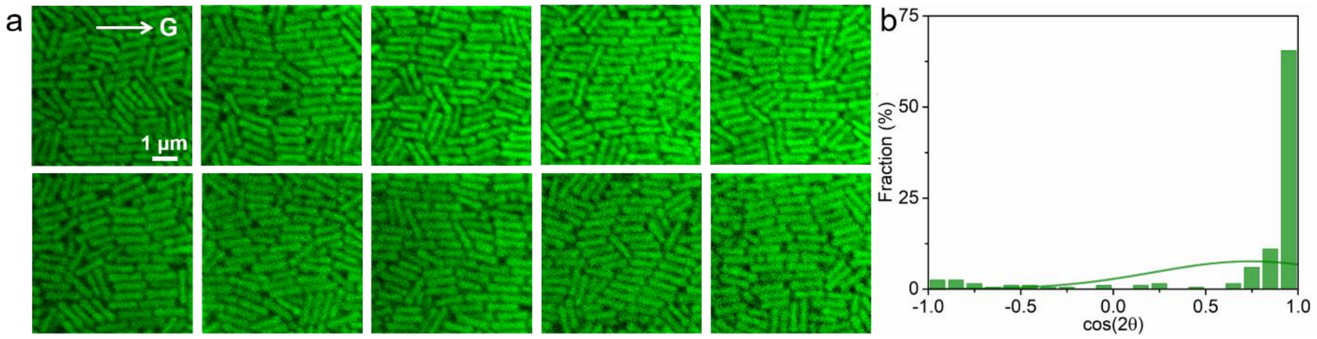

Supplementary Figure 4. The measurement of S_{2d}. (a) 10 representative confocal microscopy images of N2 phase assembled from DBCs with L_e = 400 nm, L_c = 715 nm, D_e = 280 nm, and D_c = 190 nm. (b) $\cos(2\theta)$ distribution is obtained by measuring 200 DBCs. The arrow represents the direction of gravity.

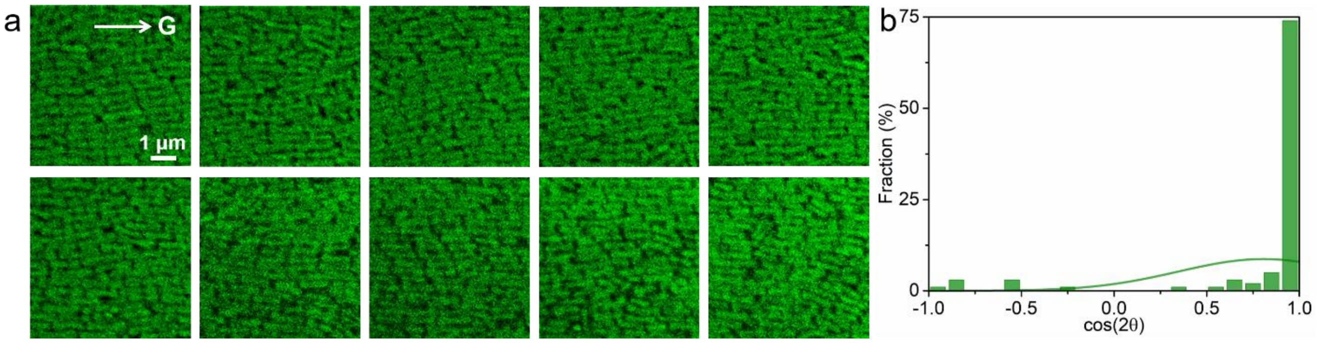

Supplementary Figure 5. The measurement of S_{2d}. (a) 10 representative confocal microscopy images of N1 phase assembled from DBCs with L_e = 515 nm, L_c = 520 nm, D_e = 235 nm, and D_c = 155 nm. (b) $\cos(2\theta)$ distribution is obtained by measuring 200 DBCs. The arrow represents the direction of gravity.

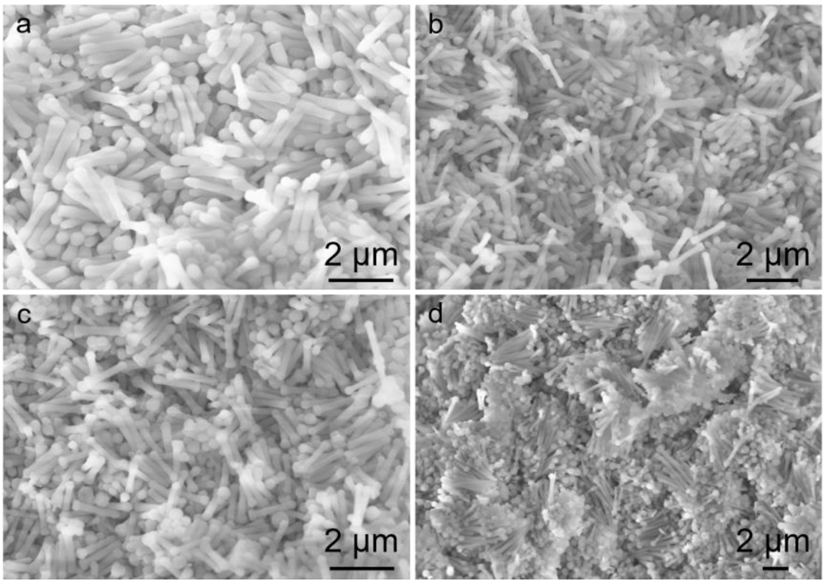

Supplementary Figure 6. BP III phase of DBCs with two very short e-blocks. SEM images of BP III phase of (**a**) DBC with L_e = 460 nm, L_c = 1145 nm, D_e = 335 nm, and D_c = 255 nm, (**b**) DBC with L_e = 200 nm, L_c = 960 nm, D_e = 315 nm, and D_c = 200 nm, (**c**) DBC with L_e = 165 nm, L_c = 1010 nm, D_e = 335 nm, and D_c = 205 nm, and (**d**) DBC with L_e = 280 nm, L_c = 3360 nm, D_e = 395 nm, and D_c = 245 nm.

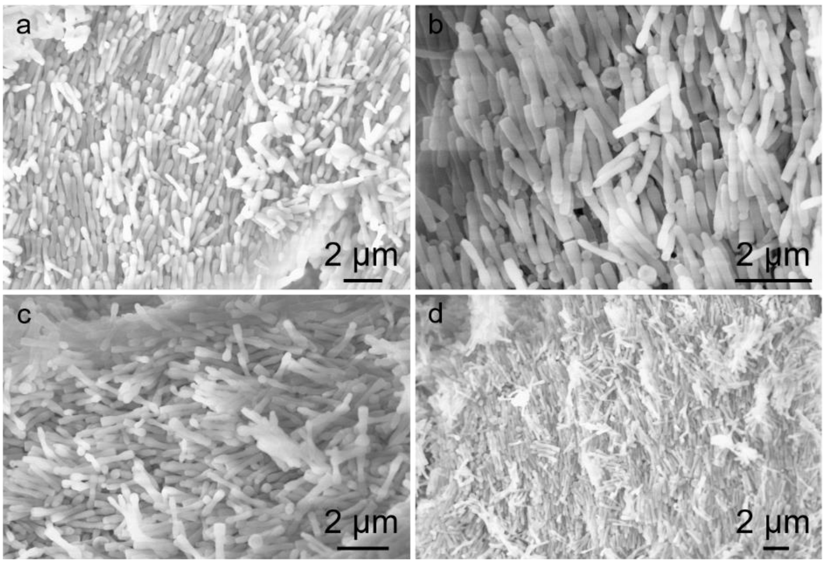

Supplementary Figure 7. N2 phase of DBCs with two short e-blocks. SEM images of N2 phase of (**a**) DBC with L_e = 460 nm, L_c = 700 nm, D_e = 300 nm, and D_c = 205 nm, (**b**) DBC with L_e = 475 nm, L_c = 650 nm, D_e = 280 nm, and D_c = 200 nm, (**c**) DBC with L_e = 350 nm, L_c = 790 nm, D_e = 295 nm, and D_c = 195 nm, and (**d**) DBC with L_e = 370 nm, L_c = 900 nm, D_e = 240 nm, and D_c = 160 nm.

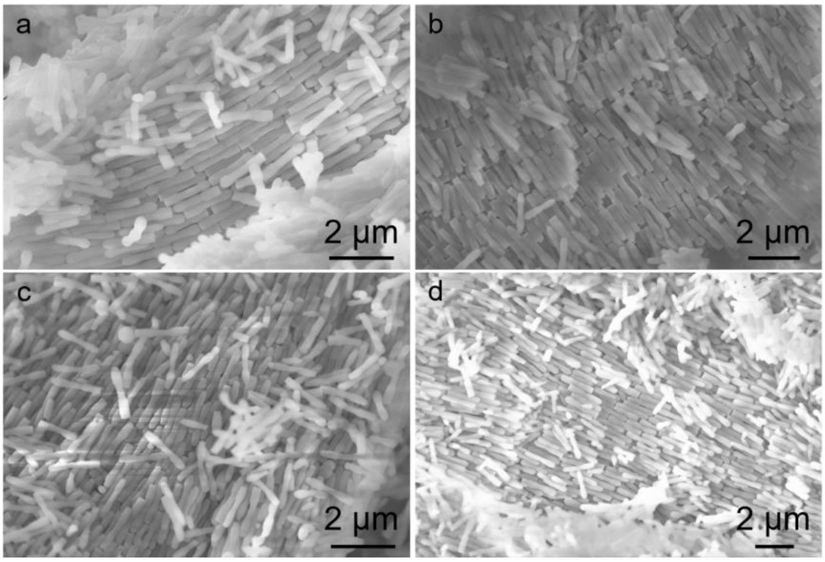

Supplementary Figure 8. N1 phase of DBCs with two intermediate e-blocks. SEM images of N1 phase of (**a**) DBC with L_e = 475 nm, L_c = 570 nm, D_e = 300 nm, and D_c = 240 nm, (**b**) DBC with L_e = 525 nm, L_c = 525 nm, D_e = 300 nm, and D_c = 245 nm, (**c**) DBC with L_e = 515 nm, L_c = 520 nm, D_e = 235 nm, and D_c = 155 nm, and (**d**) DBC with L_e = 505 nm, L_c = 395 nm, D_e = 275 nm, and D_c = 205 nm.

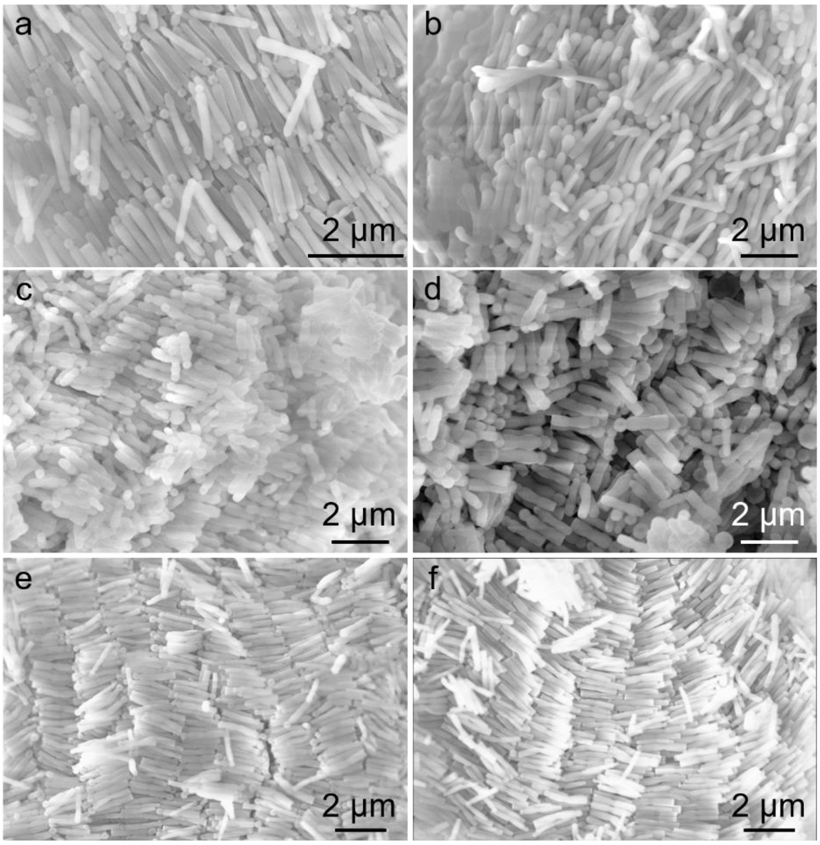

Supplementary Figure 9. SmA phase of DBCs with rod-like shape. SEM images of SmA phase of (**a**) DBC with L_e = 505 nm, L_c = 480 nm, D_e = 190 nm, and D_c = 175 nm, (**b**) DBC with L_e = 425 nm, L_c = 1455 nm, D_e = 235 nm, and D_c = 220 nm, (**c**) DBC with L_e = 505 nm, L_c = 280 nm, D_e = 340 nm, and D_c = 255 nm, (**d**) DBC with L_e = 565 nm, L_c = 400 nm, D_e = 330 nm, and D_c = 240 nm, (**e**) DBC with L_e = 660 nm, L_c = 115 nm, D_e = 225 nm, and D_c = 125 nm, and (**f**) DBC with L_e = 755 nm, L_c = 155 nm, D_e = 205 nm, and D_c = 140 nm.

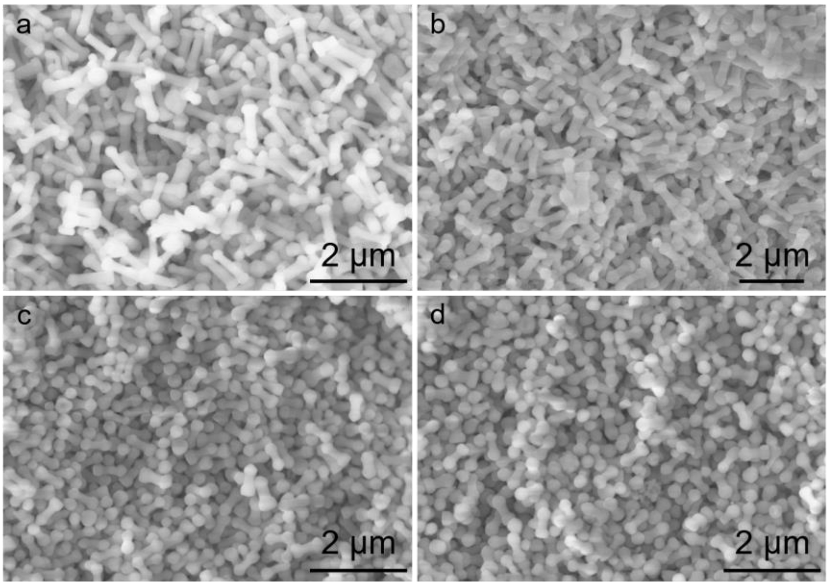

Supplementary Figure 10. Isotropic phase of DBCs with large R_D. SEM images of isotropic phase of (**a**) DBC with L_e = 170 nm, L_c = 560 nm, D_e = 295 nm, and D_c = 180 nm, (**b**) DBC with L_e = 175 nm, L_c = 555 nm, D_e = 425 nm, and D_c = 265 nm, (**c**) DBC with L_e = 145 nm, L_c = 295 nm, D_e = 275 nm, and D_c = 165 nm, and (**d**) DBC with L_e = 145 nm, L_c = 215 nm, D_e = 250 nm, and D_c = 160 nm.

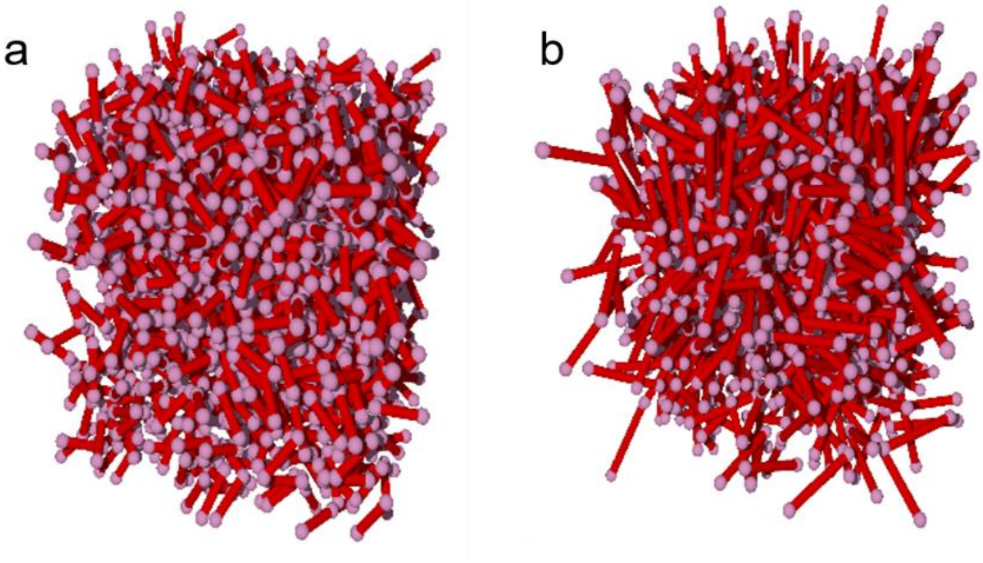

Supplementary Figure 11. Isotropic phase observed by simulation. DBCs with (**a**) R_D = 1.6, R_L = 0.7 and (**b**) R_D = 1.6, R_L = 0.3.

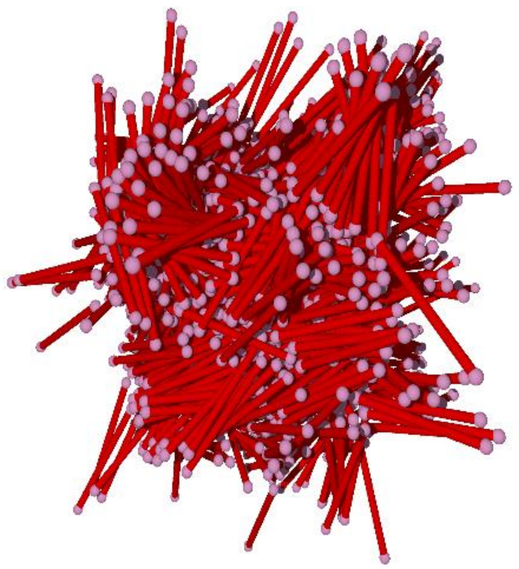

Supplementary Figure 12. BP III phase observed by simulation. DBC with R_D = 1.5 and R_L = 0.12.

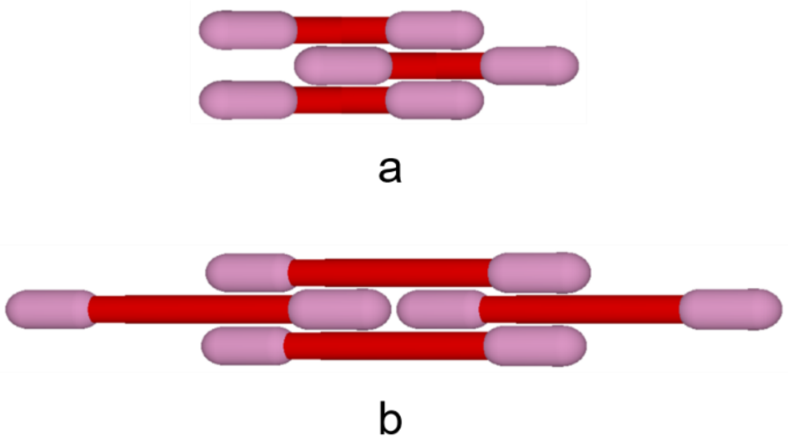

Supplementary Figure 13. Schematic illustration of one-lock and two-lock mechanism. (**a**) In the one-lock mechanism, the length of e-block is comparable to c-block; (**b**) c-block is twice the length of e-block in the two-lock mechanism.

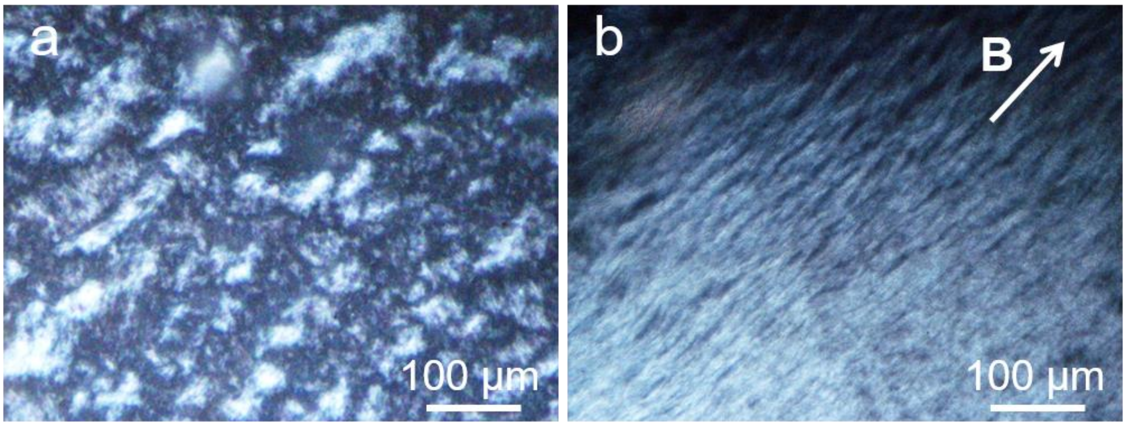

Supplementary Figure 14. The manipulation of LC phases by a magnetic field. POM images of phases formed from DBCs with L_e = 160 nm, L_c = 1660 nm, D_e = 315 nm, and D_c = 240 nm (**a**) before the application of a magnetic field of 5 T (BP III phase) and (**b**) after the application of a magnetic field of 5 T (nematic phase).
