# Supplementary material for: Liquid-crystalline behavior on dumbbell-shaped colloids and the observation of chiral blue phases
Source: Nat Commun. 2022 Sep 22;13:5549. doi: 10.1038/s41467-022-33125-y (PMC9500018; doi:10.1038/s41467-022-33125-y)
Supplement: Supplementary file 3 — Description of Additional Supplementary Files [file 41467_2022_33125_MOESM3_ESM.docx]

**Description of Additional Supplementary Files**

Title: Supplementary Video 1.

Description: Confocal microscopy images of BP phase assembled from DBCs with *L*_e_ = 160 nm, *L*_c_ = 1660 nm, *D*_e_ = 315 nm, and *D*_c_ = 240 nm. The distance between adjacent images is 300 nm. The observation is from the inner glass surface to the deeper part. There are three cycles for each video.

Title: Supplementary Video 2.

Description: Confocal microscopy images of BP phase assembled from DBCs with *L*_e_ = 310 nm, *L*_c_ = 3200 nm, *D*_e_ = 550 nm, and *D*_c_ = 470 nm. The distance between adjacent images is 300 nm. The observation is from the inner glass surface to the deeper part. There are three cycles for each video.
